# Extravasation of radiographic contrast material and compartment syndrome in the hand: a case report

**DOI:** 10.1186/1757-7241-19-9

**Published:** 2011-02-04

**Authors:** Tomas Belzunegui, Clint Jean Louis, Laura Torrededia, Julio Oteiza

**Affiliations:** 1 Emergency department. Hospital de Navarra. Pamplona. Navarra. Pamplona. Navarra. Spain; 2 Department of Orthopaedics and Traumatology. Hospital de Navarra. Pamplona. Navarra. Spain; 3 Department of Internal Medicine. Hospital de Navarra. Pamplona. Navarra. Spain Irunlarrea, s/n. 31007 Pamplona. Navarra. Spain

## Abstract

Radiocontrast agents are a type of medical contrast material used to improve the visibility of internal bodily structures in X-ray based imaging techniques such as computed tomography (CT) or radiography. Radiocontrast agents are typically iodine or barium compounds.

Extravasation of contrast is a possible complication of imaging studies performed with contrasts. Most extravasations cause minimal swelling or erythema, however, skin necrosis, ulceration and compartment syndrome may occur with extravasation of large volumes of contrast.

A case report is presented in which significant extravasation of contrast was caused while injecting the contrast intravenously into the back of the hand of a 50 year old patient during computed tomography. The patient was undergoing chemotherapy. The patient developed a compartment syndrome and a fasciotomy was required. Treatment options are outlined and emphasis is made on prevention of this iatrogenic complication.

Some of the preventive measures to avoid these complications include use of non-ionic contrast (low osmolarity), careful choice of the site of intravenous administration, and close monitoring of the patient during injection of contrast to minimize or prevent extravasation injuries. Clear information to patients and prompt recognition of the complication can allow for other non-surgical treatment options than the one required in this case.

## Background

Subcutaneous extravasation is a known complication of intravenous administration of iodinated contrast[1]. Various studies consider the rate of extravasation during CT in figures ranging from 0.03% - 0.17%[2-4]. With the systematic use of mechanical injectors, different studies have shown increasing rates of extravasation with figures ranging from 0.25% to 0.9%[5]. The clinical experience is very variable. Most cases of subcutaneous extravasastion occur due to small volumes of extravasation of contrast causing pain, minimum swelling and localized erythema, that is rapidly decreased[1]. If larger volumes are extravasated, extensive tissue and skin necrosis may occur[1,3].

Compartment syndrome located in the hand may also be associated with extravasation of large volumes of contrast[6,7]. We present a patient who developed a compartment syndrome in her right hand after extravasation of contrast while performing a CT scan.

## Case presentation

A 50 year old woman, diagnosed with stage IIB non small cell lung carcinoma, who had undergone surgery, and previous contrast thoracoabdominal CT scans to detect tumor recurrence or metastasis was programmed for another contrast enhanced CT scan to monitor her disease. She was currently under chemotherapy.

Approximately 100 ml of non-ionic iodinated contrast was extravasated (Optiray UltraJet 350 mg/ml; Mallinkrodt, St Louis, Missouri) after injection via a rapid infusion pump (Optivantage DH; Liebel-Flarsheim Company, Cincinnati, Ohio) on the dorsum of her right hand [8,9].

At the start of the injection the patient experienced swelling and severe pain in the hand, but but did not notify the personnel responsible for the test. No contrast was visible in the thoraco-abdominal images. Local ice and analgesic treatment was recommended and the patient was sent home. About five hours after the scan, given increasing pain and swelling of the hand, the patient presented at the emergency department.

**Figure 1:**
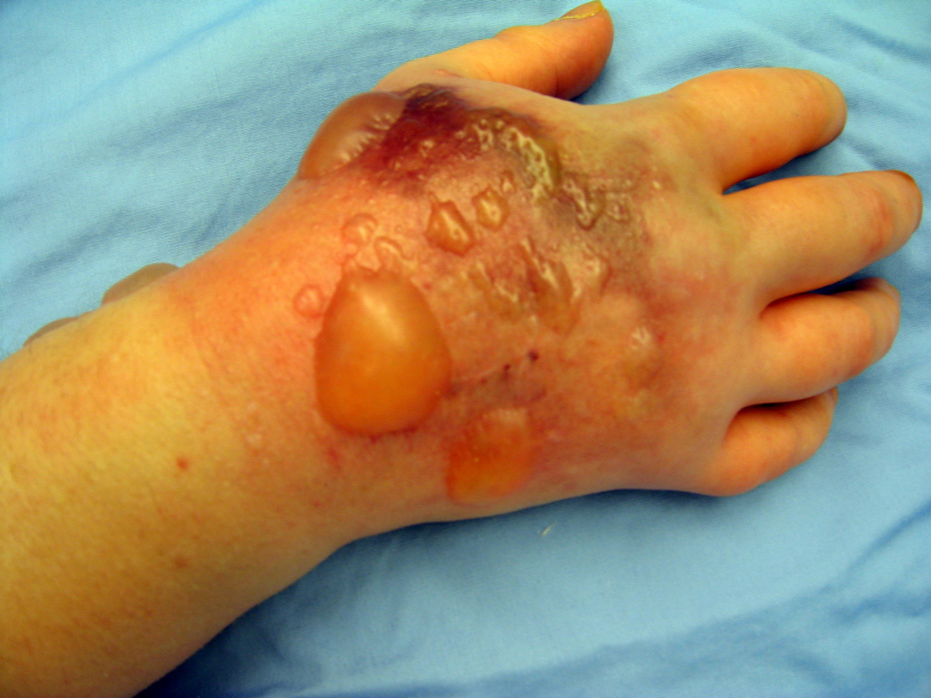
**Image showing the right hand of the patient; tissue tension, global swelling, paleness, and blisters in the dorsal region can be observed**.

Physical examination showed a pale, tense and swollen hand, with blisters on the back and loss of sensation. Capillary refill was increased and the patient was unable to move her right fingers, and any attempt to do so was extremely painful (Figure 1).

Conservative measures (ice, elevation of the forearm, intravenous administration of corticosteroids and analgesic treatment), did not improve the symptoms.

**Figure 2:**
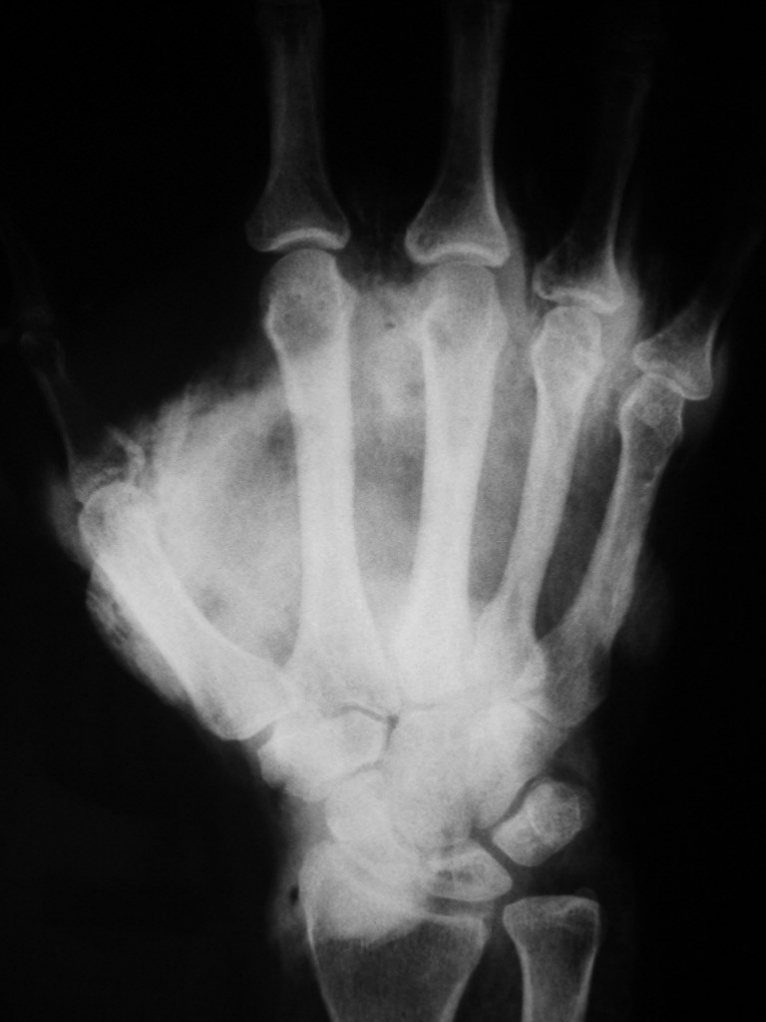
**Simple X-ray of the hand that shows a considerable accumulation of extravascular contrast**.

Plain X-rays of the hand showed a significant accumulation of contrast within the extravascular space (Figure 2).

**Figure 3:**
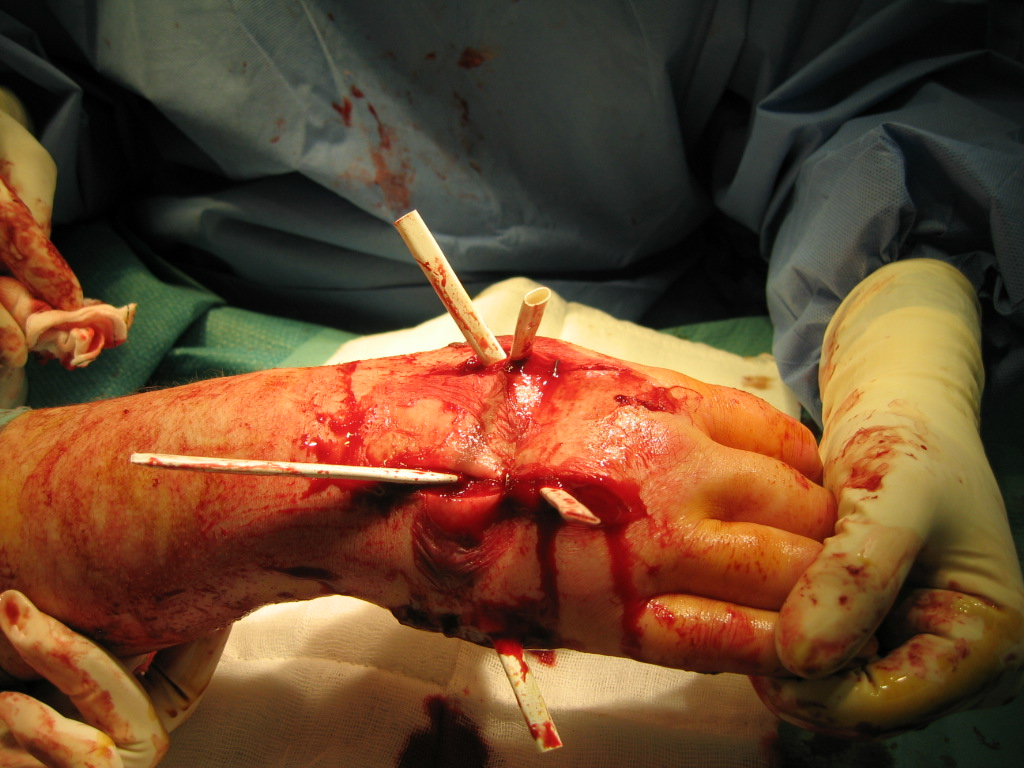
**Intraoperative image of the dorsal region of the right hand of the patient after removal of the hematoma, aspiration of iodinated contrast and fasciotomy**. Please note the placement of drains

Compartment syndrome was diagnosed, and 6 hours after the injection of contrast the patient was admitted for surgery where longitudinal incisions through the 2^nd ^and 4^th ^metacarpal ridges on the dorsum of the hand were performed. The hematoma was evacuated by pressure. Infiltration of a transparent material in viscous-liquid form (iodinated contrast) within the subcutaneous tissue was observed. The four interosseous compartments and that of the thumb adductor were released. Fasciotomy of thenar and hypothenar eminences was performed and the carpal annular ligament was released, observing very swollen interosseous muscles and no macroscopic evidence of necrosis. Six Penrose drains were left and the edges of the surgical wound were closed with staples (Figure 3).

The day after surgery, swelling and pain had significantly decreased, and capillary refill had improved. There were no new blisters. Three days after surgery the drains were removed, and 7 days after surgery the patient had recovered sensation and motor function in the hand. In the follow-up on day 30 after surgery the patient had fully recovered mobility and sensation. The surgical wounds had healed with full recovery of hand function.

## Discussion

Compartment syndrome is a complex of symptoms caused by increasing pressure of soft tissues within a confined space that threatens blood circulation and the functions of the structures found within in that space. In the hand, the most common causes of compartment syndrome are fractures, crushing and other soft tissue injuries such as burns, arterial injuries, snake bites and infections[10].

Compartment syndromes of the hand or forearm secondary to extravasation of contrast have been reported[6,7,11]. Important factors affecting the severity of extravasation injuries include osmolality, ionic or non-ionic nature of the compound, and the nature and volume of the extravasation [6,11].

Causes for extravasation may depend on the technique (injection of large volumes or at a fast rate through the infusion pump) or on the patients characteristics (unable to comunicate as in unconsciousness, fragile blood vessels especially in elderly patients and patients under chemotherapy).

The main reasons involved in the increase of accidental extravasation of contrast volumes exceeding 50 ml are the use of rapid infusion pumps and the increase in the use of CT scans in monitoring cancer patients[1,2]. These patients, often under chemotherapy, should be particularly monitored at the time of contrast infusion, especially if the IV line is on the dorsum of the hand, since chemotherapy induces fragility of the vein wall which can lead to the vessels rupture when starting a rapid infusion.

It is interesting to note the development and implementation in daily practice of devices that detect early contrast extravasation based on the change of skin impedance[12].

Other aspects to consider in patients when performing contrast enhanced CT scans are associated arterial or venous insufficiency, poor lymphatic drainage, low muscle mass and subcutaneous tissue atrophy [4,5].

The clinical manifestations of extravasation of contrast can range from mild redness and swelling of the tissue to necrosis associated with progressive edema of the skin and ulceration. Occasionally, necrosis may occur, resulting, in the case of the hand, in retraction of flexo-extensor muscles and consequent loss of hand function[1,4,5,7].

The vast majority of extravasations of contrast are of small volumes. The large volume extravasations occur mainly when using rapid infusion pumps. In our case, we used a rapid infuser, and the patient, in spite of feeling pain, didn't warn the medical personnel immediately, while the 100 ml of contrast passed into the extravascular space.

There is no general agreement regarding the best approach for the management of extravasation. The elevation of the limb is often useful to reduce edema and cooling the injection site with ice packs is very useful in limiting inflammation.

The injection of hyaluronidase (enzyme that breaks down the connective tissue and helps the absorption of extravasated drugs by the vascular and lymphatic systems) has also been recommended for patients with large extravasation volumes. Corticosteroids, vasodilators, and a variety of other drugs have also been proposed for the treatment of extravasation, but most studies have not shown its efficacy[4,5,11].

Most surgeons believe that a large proportion of injuries caused by extravasation heal without surgery and recommend a conservative approach[10].

However, urgent surgical drainage and aspiration of contrast performed in the first 6 hours has been effective when a compartment syndrome has occurred in cases of large extravasations [13].

In our case we opted for an emergency procedure taking into account the significant swelling of the hand and the threat that could result in delaying dorsal emergency fasciotomy and carpal tunnel release. The reviewed literature agrees that this procedure should be performed as soon as possible and ideally within the first 6 hours in order to relieve neurovascular compromise [1,6,7].

It is very important to thoroughly document all incidents occurring during imaging scans with iodinated contrasts, as this allows us to know the incidence and severity of symptoms, helps to determine whether the infusion was adapted to the established standards, and is the cornerstone of medico-legal defense should they occur[3].

In our case the errors detected included an inadequate intravenous access site in a patient with high risk of vessel rupture, the patient was not insisted upon to report on any abnormal symptom arising, and the patient was not remitted to the emergency department immediately.

The risk of extravasation can be reduced by the use of non-ionic contrasts of lower osmolarity which produce less direct tissue damage than ionic contrasts of higher osmolarity. Direct supervision of infusion pumps or the use of devices that can detect early extravasation through impedance are useful. Larger veins found at the antecubital fossa are recommended sites for intraveous access and appropiate catheter gauge should be considered to withstand infusions. Clear instructions should be given to the patient to report of any pain or any discomfort at the site of injection.

## Conclusions

Contrast extravasation is a rare complication of imaging studies. The extravasation of large volumes of contrast sometimes occurs when using automatic infusers and can lead to serious consequences, especially if extravasation occurs in the hand. Close monitoring of pump based infusion of contrast in the back of the hand is essential in cancer patients. If compartment syndrome develops as a result of contrast extravasation, emergency dorsal fasciotomy and carpal tunnel release must be performed within the first 6 hours to relieve neurovascular compromise. Simple measures can be employed to prevent a serious iatrogenic complication.

## Consent

Written informed consent was obtained from the patient for publication of this case report and any accompanying images. A copy of the written consent is available for review by the Editor-in-Chief of this journal.

## Competing interests

The authors declare that they have no competing interests.

## Authors' contributions

All authors have made substantive contributions to the study, and all authors endorse the data and conclusions.

All authors read and approved the manuscript.
